# Sex affects *N*-homocysteinylation at lysine residue 212 of albumin in mice

**DOI:** 10.1038/s41598-019-38784-4

**Published:** 2019-02-25

**Authors:** Marta Sikora, Łukasz Marczak, Joanna Perła-Kajan, Hieronim Jakubowski

**Affiliations:** 1 European Centre for Bioinformatics and Genomics, Institute of Bioorganic Chemistry, Polish Academy of Sciences, Poznań, Poland; 2 Department of Biochemistry and Biotechnology, University of Life Sciences, Poznań, Poland; 3 Department of Microbiology, Biochemistry and Molecular Genetics, Rutgers-New Jersey Medical School, International Center for Public Health, Newark, NJ USA

## Abstract

The modification of protein lysine residues by the thioester homocysteine (Hcy)-thiolactone has been implicated in cardiovascular and neurodegenerative diseases. However, only a handful of proteins carrying Hcy on specific lysine residues have been identified and quantified in humans or animals. In the present work, we developed a liquid chromatography/mass spectrometry targeted assay, based on multiple reaction monitoring, for quantification of *N*-Hcy-Lys212 (K212Hcy) and *N*-Hcy-Lys525 (K525Hcy) sites in serum albumin in mice. Using this assay, we found that female (n = 20) and male (n = 13) *Cbs*^−/−^ mice had significantly elevated levels of K212Hcy and K525Hcy modifications in serum albumin relative to their female (n = 19) and male (n = 17) *Cbs*^+/−^ littermates. There was significantly more K212Hcy modification in *Cbs*^−/−^ males than in *Cbs*^−/−^ females (5.78 ± 4.21 *vs*. 3.15 ± 1.38 units, *P* = 0.023). Higher K212Hcy levels in males than in females were observed also in *Cbs*^+/−^ mice (2.72 ± 0.81 *vs*. 1.89 ± 1.07 units, *P* = 0.008). In contrast, levels of the K525Hcy albumin modification were similar between males and females, both in *Cbs*^−/−^ and *Cbs*^+/−^ mice. These findings suggest that the sex-specific K212Hcy modification in albumin might have an important biological function in mice that is not affected by the *Cbs* genotype.

## Introduction

The sulfur-containing amino acid homocysteine (Hcy) is an intermediate in the metabolic pathways of two canonical amino acids that participate in the genetic code: methionine (Met) and cysteine (Cys). Hcy levels are regulated by re-methylation to Met, catalyzed by Met synthase (with methyltetrahydrofolate cofactor provided by the MTHFR enzyme) and betaine-Hcy methyltransferase, as well as by transsulfuration to cysteine catalyzed by cystathionine β-synthase (CBS) and cystathionine γ-lyase^1^. Although Hcy, in contrast to Met and Cys, is a non-coded amino acid that cannot participate in canonical protein biosynthesis, it can be incorporated into proteins *via* distinct mechanisms^2–5^. In one mechanism Hcy is first erroneously selected in place of Met by methionyl-tRNA synthetase and metabolized to Hcy-thiolactone^3,5^. Like other biological thioesters (*e*.*g*., acetyl-coenzyme A^6^), Hcy-thiolactone is chemically reactive and modifies protein lysine residues generating KHcy-proteins in a process called *N-*homocysteinylation^7^. *N-*homocysteinylation alters protein’s structure/function and contributes to a variety of pathologies associated with genetic or dietary hyperhomocysteinemia (HHcy)^3,5^.

The major cause of genetic HHcy in humans is CBS deficiency with world-wide incidence of 1:344,000^1^ that in some countries can be as high as 1:65,000 (Ireland)^8^, 1:1,800 (Qatar)^9^, or even 1:240 (an Austronesian Taiwanese Tao tribe)^10^. CBS deficiency is associated with mental retardation, ectopia lentis, osteoporosis, and vascular complications (thromboembolism), which are the major cause of morbidity and mortality^1^. Hcy-thiolactone and *N*-Hcy-protein levels are elevated in CBS-deficiency, both in humans and mice^11–14^. In CBS-deficient patients, *N*-Hcy-protein accumulation has been linked to an autoimmune response and atherothrombosis^3,5^.

We have previously identified K525Hcy^15^, K212Hcy, and K137Hcy in human serum albumin^16,17^, as well as αK562Hcy, βK344Hcy, and γK385Hcy in human fibrinogen^18^ from CBS-deficient patients. Although protein *N-*homocysteinylation is increased in mouse models of HHcy, individual mouse *N*-Hcy-proteins and their sites of Hcy modification have not yet been identified *in vivo*.

The objective of the present study was to identify and quantify KHcy residues in mouse serum albumin and to study how sex, age, total Hcy (tHcy), and *Cbs* genotype affect mouse albumin KHcy modification *in vivo* using *Tg-I287T Cbs*^−/−^ and *Tg-I287T Cbs*^+/−^ mice.

## Results

### Identification of KHcy sites in mouse albumin modified with Hcy-thiolactone *in vitro*

**Figure 1:**
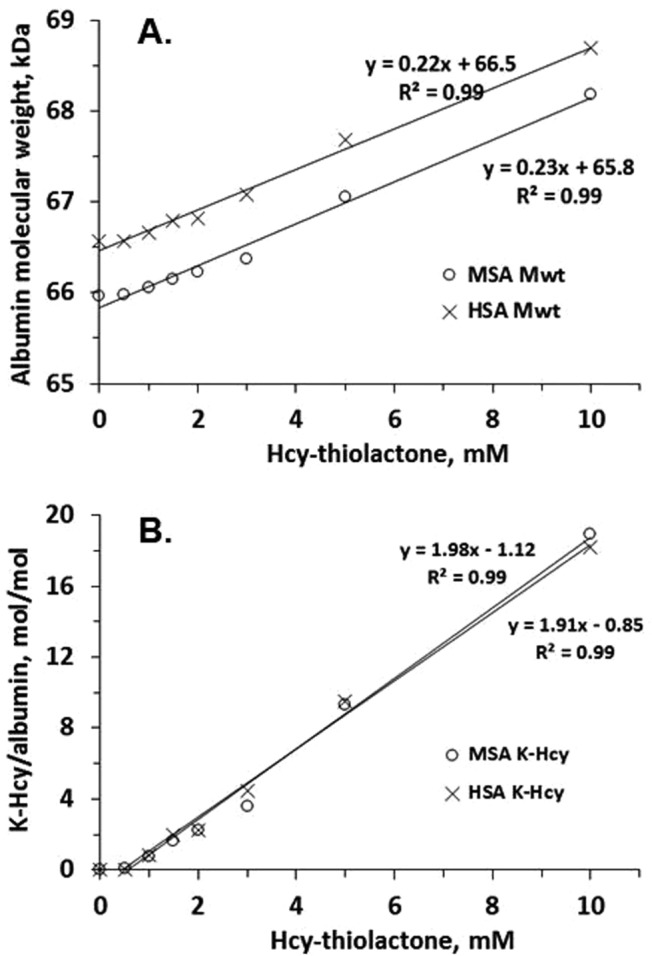
Relationships between Hcy-thiolactone concentration and molecular weight of (**A**) and number of KHcy residues (**B**) in mouse (MSA) and human (HSA) albumin.

We modified mouse serum albumin *in vitro* with increasing concentrations of Hcy-thiolactone and analysed the changes in molecular weight of albumin using electrospray ionization mass spectrometry (ESI MS). There was a linear increase in the molecular weight, from 66,565 Da for unmodified albumin to 68,695 Da for the modified KHcy-albumin (Fig. 1). The 2,140 Da increase in molecular weight indicates incorporation of *ca*. [2,140/119.2] = 18 moles of Hcy per mol of albumin for the highest Hcy-thiolactone concentration used (Fig. 1). This suggests that at least 18 out of 51 lysine residues (35.3%) in mouse serum albumin were modified under these conditions. Similar relationships were observed for the KHcy modification of human serum albumin (Fig. 1).

Using liquid chromatography with tandem mass spectrometry (LC/MS-MS), we identified twenty eight KHcy residues in mouse serum albumin modified *in vitro* with Hcy-thiolactone (Table 1).

**Table 1:** KHcy-peptides identified in tryptic digests *in vitro*-modified mouse KHcy-albumin.

	Sequence	Area	m/z [Da]	Range	Modification
1	R.AFKAWAVAR.L	4.04E11	597.32	210–218	K212Hcy
2	K.KQTALAELVK.H	3.44E11	637.86	525–534	K525Hcy
3	K.LQTCCDKPLLK.K	3.11E11	517.26	275–286	K281Hcy
4	R.YTQKAPQVSTPTLVEAAR.N	2.53E11	1067.56	411–428	K414Hcy
5	K.LQTCCDKPLLKK.A	1.59E11	559.95	275–286	K281/285Hcy
6	K.TPVSEHVTKCCSGSLVER.R	1.57E11	1110.51	467–484	K475Hcy
7	R.RPCFSALTVDETYVPKEFK.A	1.46E11	821.07	484–503	K500Hcy
8	R.VGTKCCTLPEDQR.L	1.24E11	869.39	433–445	K436Hcy
9	K.EKALVSSVR.Q	1.09E11	581.82	187–195	K188Hcy
10	K.NLVKTNCDLYEK.L	1.06E11	835.90	386–397	K389Hcy
11	R.VCLLHEKTPVSEHVTK.C	1.01E11	684.35	460–475	K466Hcy
12	K.HKPKATAEQLK.T	8.59E10	712.89	235–245	K236/238Hcy
13	K.KYEATLEK.C	8.57E10	578.29	352–359	K352Hcy
14	K.AADKDTCFSTEGPNLVTR.C	8.27E10	1078.49	561–578	K564Hcy
15	K.AETFTFHSDICTLPEKEK.Q	8.24E10	776.36	504–521	K519Hcy
16	K.LATDLTKVNK.E	6.94E10	638.85	234–243	K240Hcy
17	K.EFKAETFTFHSDICTLPEK.Q	6.09E10	1237.57	501–519	K503Hcy
18	K.LVQEVTDFAKTCVADESAANCDK.S	5.58E10	915.74	42–64	K51Hcy
19	K.LDGVKEKALVSSVR.Q	5.55E10	924.99	182–195	K186Hcy, K188Hcy
20	K.QIKKQTALAELVK.H	5.14E10	909.50	522–534	K524Hcy, K525Hcy
21	R.ENYGELADCCTKQEPER.N	4.36E10	1136.97	82–98	K93Hcy
22	K.CSSMQKFGER.A	4.07E10	702.30	200–209	K206Hcy
23	K.LQTCCDKPLLKK.A	3.67E10	926.45	275–286	K281Hcy, K285Kcy
24	K.TCVADESAANCDKSLHTLFGDK.L	2.89E10	654.04	52–73	K64Hcy
25	K.VNKECCHGDLLECADDR.A	2.01E10	1132.96	241–257	K243Hcy
26	K.CSYDEHAKLVQEVTDFAK.T	1.98E10	772.02	34–51	K41Hcy
27	K.HKPKATAEQLK.T	1.93E10	533.61	235–245	K236Hcy, K238Hcy
28	K.SLHTLFGDKLCAIPNLR.E	1.78E10	1065.05	65–81	K73Hcy

Two of those modifications, K212Hcy and K525Hcy, present in ^525^K^Hcy^QTALAELVK^534^ (m/z 637.8) and ^210^AFK^Hcy^AWAVAR^218^ (m/z 597.3) peptides, were the most abundant.

**Figure 2:**
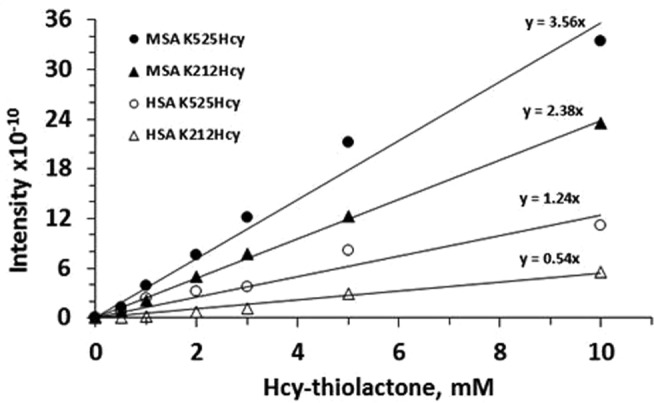
Relationships between Hcy-thiolactone concentration and the magnitude of K212Hcy and K525Hcy modifications in mouse (MSA) and human (HSA) serum albumins.

Quantification of K212Hcy and K525Hcy modifications in the *in vitro*-modified human and mouse albumins showed that there was a linear relationship between Hcy-thiolactone concentration and the magnitude of these modifications (Fig. 2). Notably, the K212 and K525 residues were more susceptible to modification in the mouse than in human albumin, as indicated by the greater slopes of the ‘Intensity *vs*. Hcy-thiolactone’ plots for the mouse K212Hcy (4.4-fold) and K525Hcy (2.9-fold) residues in Fig. 2.

### Identification/quantification of albumin KHcy modifications in mouse plasma *in vivo*

**Figure 3:**
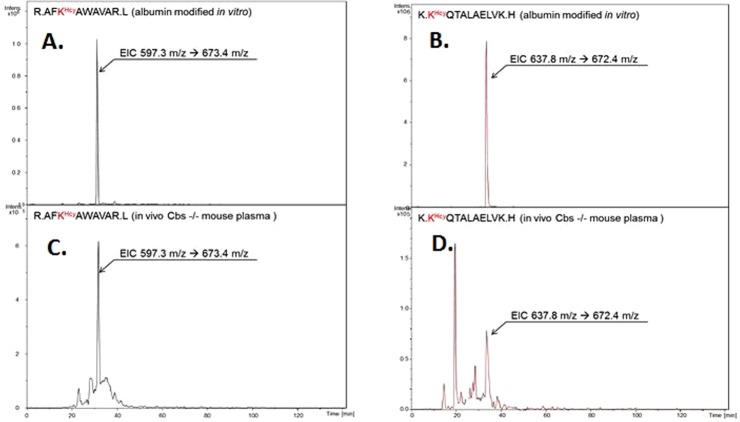
Extracted ion chromatograms of peptides ^210^AFK^Hcy^AWAVAR^218^ (m/z 597.3) and ^525^K^Hcy^QTALAELVK^534^ (m/z 637.8) from a tryptic digests of mouse albumin *N*-homocysteinylated with Hcy-thiolactone *in vitro* (**A**,**B**) and of plasma from *Cbs*^−/−^ mouse (**C**,**D**).

Having established the masses of KHcy-peptides from tryptic digests of the *in vitro*-prepared mouse KHcy-albumin, we quantified these modifications directly in tryptic digests of mouse plasma. Examples of LC/MS-MS MRM analyses and extracted ion chromatograms for KHcy albumin modifications identified in mouse plasma *in vivo* and in *in vitro*-prepared mouse KHcy-albumin are shown in Fig. 3. We found that albumin K212Hcy and K525Hcy modifications, present in ^210^AFK^Hcy^AWAVAR^218^ (m/z 597.3) and ^525^K^Hcy^QTALAELVK^534^ (m/z 637.8) peptides, respectively, were detectable in each mouse plasma sample. Other KHcy modifications were detectable in some samples, most likely because of their low abundance.

To examine determinants of K212Hcy and K525Hcy modifications we quantified ^210^AFK^Hcy^AWAVAR^218^ (597.3 m/z) and ^525^K^Hcy^QTALAELVK^534^ (637.8 m/z) peptides in tryptic digests of plasma from HHcy *Cbs*^−/−^ mice (median plasma tHcy 200.8 µM, range 47.4 to 346 µM, n = 23), which also have elevated levels of KHcy-protein (16.6 ± 4.1 µM)^12^ and from control *Cbs*^+/−^ mice (median plasma tHcy 6.4 µM, range 1.2 to 11.2 µM, n = 12), which have low levels of KHcy-protein (2.62 ± 1.73 µM)^12^.

Mean levels of K212Hcy and K525Hcy modifications were significantly higher (~2-fold) in *Tg-I287T Cbs*^−/−^ mice compared with *Tg-I287T Cbs*^+/−^ littermates, both in females and in males (Table 2). Notably, the levels of K212Hcy modification were significantly higher in male than in female mice. In contrast, the levels of K525Hcy modification were similar in females and males.

**Table 2:** Effects of sex and *Cbs* genotype on K525Hcy and K212Hcy modifications in mouse albumin.

Sex (n)	Genotype (n)	K525Hcy	K212Hcy
		arbitrary units x 10^−5^	
Female (39)	*Tg-I287T Cbs*^−/−^ (20)	1.42 ± 0.67	3.15 ± 1.38
	*Tg-I287T Cbs*^+/−^ (19)	0.87 ± 0.50	1.89 ± 1.07
	*P* value, *Cbs*^−/−^ *vs*. *Cbs*^+/−^	0.005	0.006
Male (30)	*Tg-I287T Cbs*^−/−^ (13)	1.62 ± 1.01	5.78 ± 4.21
	*Tg-I287T Cbs*^+/−^ (17)	0.92 ± 0.41	2.72 ± 0.81
	*P* value, *Cbs*^−/−^ *vs*. *Cbs*^+/−^	0.009	0.004
*P* value, F *vs*. M	*Tg-I287T Cbs*^−/−^ (33)	0.535	0.023
	*Tg-I287T Cbs*^+/−^ (36)	0.514	0.008

**Figure 4:**
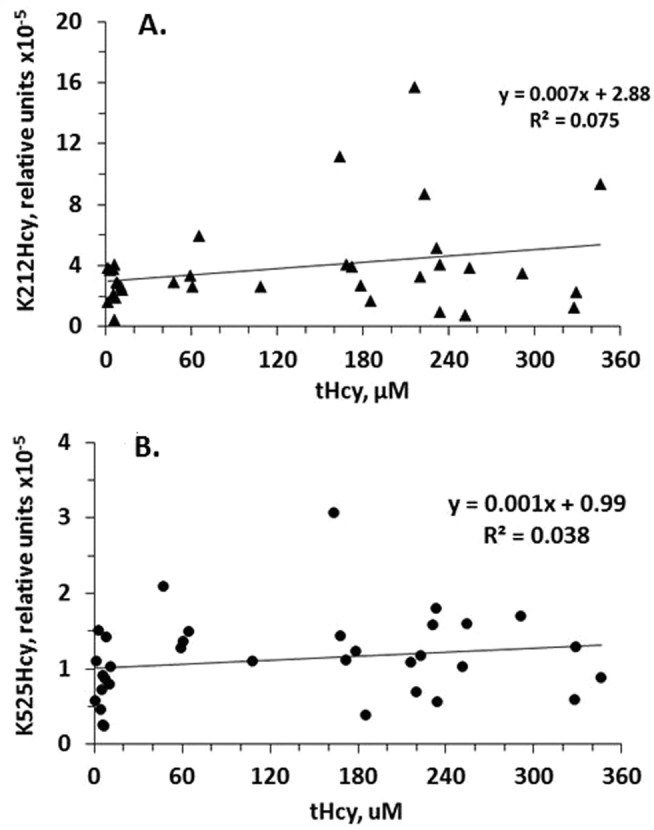
Relationships between albumin K212Hcy (**A**) and K525Hcy (**B**) modifications and tHcy in *Cbs*^+/−^ and *Cbs*^−/−^ mice.

Total Hcy explained 7.5% (Fig. 4A) and 3.8% (Fig. 4B) of the variance in K212Hcy and K525Hcy, respectively.

**Figure 5:**
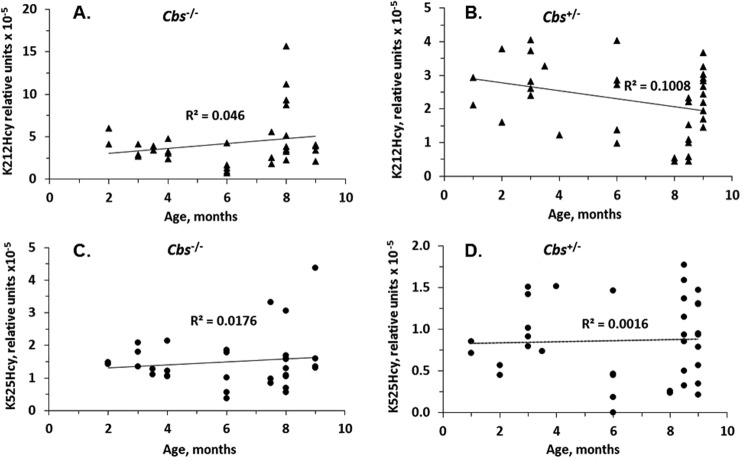
Relationships between albumin K212Hcy (**A**,**B**) and K525Hcy (**C**,**D**) modifications and age in *Cbs*^−/−^ (**A**,**C**) and *Cbs*^+/−^ (**B**,**D**) mice.

Age explained 4.6% (Fig. 5A) and 10.08% (Fig. 5B) of the variance in K212Hcy in *Tg-I287T Cbs*^−/−^ mice and their *Tg-I287T Cbs*^+/−^ littermates, respectively. In contrast, only 1.76% (Fig. 5C) and 0.16% (Fig. 5D) of the K525Hcy variance in these mice was explained by their age.

## Discussion

Since the discovery of KHcy-protein in human plasma^19^, the list of KHcy-proteins identified *in vivo* has grown to a few dozen^5^. For some of these proteins the *in vivo* sites of KHcy modifications have been identified. These include human serum albumin^15–17^, fibrinogen^18^, histones^20^ and DNA damage repair proteins^21^, rat dynein^22^, actin and E-cadherin^23^, and mouse collagen^13^. The present findings add mouse serum albumin to this list.

An unexpected finding of the present work is that the K212Hcy modification in albumin is sex-specific and is significantly higher in male than in female mice, in contrast to the K525Hcy modification, which was not affected by sex. Interestingly, the sex dependence of the K212Hcy modification was independent of the *Cbs* genotype. These findings suggest that the sex-specific K212Hcy modification in albumin is likely to play an important biological function in mice, which remains to be elucidated.

In humans, factors that affect KHcy-protein levels include the *PON1* gene variants and HHcy caused by the *CBS* or *MTHFR* gene mutations. In mice, the determinants of KHcy-protein levels include the status of genes involved in the metabolism of Hcy (*Cbs*), Hcy-thiolactone (*Pon1*, *Blmh*), or folate (*Mthfr*, *Pcft*), as well as a high methionine diet^3^. In general, KHcy-protein levels increase in HHcy and in Hcy-thiolactonase deficiencies. For instance, plasma *N*-Hcy-protein levels increase 31.4-fold in CBS-deficient patients^11^ and 8.1-fold in *Cbs*^−/−^ mice, relative to unaffected individuals^12^. Elevated KHcy-protein levels are associated with low Hcy-thiolactonase activity of PON1 in humans and Pon1 or Blmh in mice^5^.

The present study identifies *Cbs* genotype as a determinant of albumin K212Hcy and K525Hcy modifications in mice. The mass spectrometry MRM assay shows about 2-fold higher albumin K212Hcy and K525Hcy modifications in plasma of *Cbs*^−/−^ mice than in their *Cbs*^+/−^ littermates. A chemical assay used in previous studies shows 8.1-fold higher KHcy-protein levels in plasma of *Cbs*^−/−^ mice than in their *Cbs*^+/−^ littermates^12^. This suggests that the total KHcy modifications of all other plasma proteins exceed KHcy modifications of albumin in *Cbs*^−/−^ mice. As shown in the present work, age and tHcy levels explain at best up to 10% of the variation in albumin K212Hcy and K525Hcy modifications (Figs 4 and 5). Notably, the K212Hcy modification exhibits greater variation with age than the K525Hcy modification (Fig. 5), again suggesting that the K212Hcy modification in albumin is likely to play an important biological function in mice.

Quantification of *N*-homocysteinylation at K212 and K525, lysine residues most susceptible to the modification in mouse and human albumins *in vitro*, revealed a linear increase in the magnitude of these modifications with the increasing concentration of Hcy-thiolactone (Fig. 2). While total *N*-homocysteinylation (at all sites) was similar for mouse and human albumins (Fig. 1), the site-specific *N*-homocysteinylation at K212 and K525 was greater in mouse than in human albumin (Fig. 2). This suggests that K212 and K525 residues are more reactive with Hcy-thiolactone in mouse albumin than in human albumin.

The KHcy modification is conserved in serum albumins from a variety of species, from human, pig, sheep, rabbit, rat and mouse to chicken^19^. More KHcy is present in rodent albumins (0.5% to 0.9% in mice and rats) than in human albumin (0.3%)^24^. The present findings, showing that K212 and K525 have greater reactivity towards Hcy-thiolactone in mouse albumin than in human albumin (Fig. 2), provide a possible explanation for these differences.

Identification of K212Hcy and K525Hcy residues in mouse serum albumin both *in vitro* and *in vivo* strongly suggests that these modifications are formed *in vivo* as products reactions of Hcy-thiolactone with the protein lysine residues. Analogous albumin modifications occur *in vivo* in humans^15,18^, indicating the conservation of the KHcy albumin modifications between rodents and humans.

In conclusion, to the best of our knowledge, the present findings represent the first identification and quantification of KHcy modifications at specific lysine residues of albumin in mice. We identified the sex-specific K212Hcy modification in albumin that is not affected by the *Cbs* genotype. These findings suggest an important biological function for the K212Hcy modification in mice and underscore the need to identify other determinants of the KHcy modifications and elucidate their roles in health and disease.

## Methods

### Mice

*Tg-I278T Cbs*^−/−^ mice on the C57BL/B6 genetic background were kindly provided by Warren Kruger^25^. These mice express human *CBS I278T* transgene under control of the zinc-inducible metallothionein promoter, which allows one to rescue the neonatal lethality phenotype of *Cbs*^−/−^ mice by supplementing the drinking water of pregnant dams with 25 mM zinc chloride. Zinc-water is replaced by plain water after weaning at 4 weeks. Mice are fed a standard rodent diet (TD.04352, Harlan Teklad, Madison, WI). We examined 1 to 9-months-old *Tg-I278T Cbs*^−/−^ mice with severely elevated tHcy and their *Tg-I278T Cbs*^+/−^ siblings with normal tHcy levels as controls^12^. Animal procedures were approved by the Institutional Animal Care and Use Committee at the Rutgers-New Jersey Medical School. All experiments were performed in accordance with relevant guidelines and regulations.

### Preparation of mouse KHcy-albumin

KHcy-albumin was prepared by incubation of mouse serum albumin (150 μM; MilliporeSigma) with 0.01–10 mM *L*-Hcy-thiolactone·HCl (MilliporeSigma), 0.1 M sodium phosphate buffer (pH 7.4), 0.1 mM ethylenediaminetetraacetic acid (EDTA) (overnight, 37 °C).

### Trypsin digestion of KHcy-albumin and mouse plasma

KHcy-albumin was reduced with 5.5 mM dithiothreitol (5 min, 95 °C), free thiols were blocked with 11 mM iodoacetate (20 min, darkness), and digested with sequencing-grade trypsin in 50 mM NH_4_HCO_3_ (trypsin-protein ratio 1:50, overnight, 37 °C). To identify sites of KHcy modifications in mouse albumin *in vivo*, plasma from *Tg-I287T Cbs*^−/−^ and *Tg-I287T Cbs*^+/+^ mice was diluted 60-fold in 50 mM NH_4_HCO_3_, processed and trypsinized as above.

### Mass spectrometry and data analysis

#### *In vitro* assays of KHcy-albumin

Tryptic peptides from *in vitro*-prepared KHcy-albumin were analyzed using Dionex UltiMate 3000 RSLC nanoLC System connected to Q Exactive Orbitrap mass spectrometer (Thermo Fisher Scientific). The peptides were separated on Acclaim PepMap RSLC nanoViper C18 column (75 µm × 25 cm, 2 µm granulation) eluted with acetonitrile (4–60% linear gradient in 0.1% formic acid, flow rate 300 nL/min, 230 min, 30 °C). The spectrometer was operated in data-dependent MS/MS mode with survey scans acquired at resolution of 70,000 at m/z 200 in MS mode, and 17,500 at m/z 200 in MS2 mode. Spectra were recorded in the scanning range of 300–2000 m/z in the positive ion mode. Higher energy collisional dissociation (HCD) ion fragmentation was performed with normalized collision energies set to 25. All data handling was performed using Proteome Discoverer 1.4 software (Thermo Scientific). Protein identification was done using Swiss-Prot human database with a precision tolerance 10ppm for peptide masses and 0.08 Da for fragment ion masses. The KHcy modification was introduced to Mascot database prior to all searches (mass of carbamidomethylated KHcy is 174.00 Da).

#### *In vivo* analysis of albumin K212Hcy and K525Hcy modifications in mouse plasma

For multiple reaction monitoring (MRM) analysis on Ion trap MS the transitions for main KHcy peptides were 597.3 → 673.4 m/z (K212Hcy) and 637.8 m/z → 672.4 m/z (K525Hcy). Analyzes were carried out using an ESI-IonTrap (Amazon SL, Bruker Daltonics) mass spectrometer coupled with a UPLC system (nanoAQUITY, Waters). The effluent from the nanoLC column (15 cm, 75-µm-i.d. C18 column fitted with a C18 pre-column (nanoAQUITY)), was directly introduced into the Ion Trap in positive ESI mode. The column was eluted with acetonitrile a (4 to 60% gradient in 0.1% formic acid, flow rate 300 nL/min, 140 min, 30 °C). Ion trap charge control was used to control ion accumulation in the trap. For precursor ion isolation, a 3-Da window was set up, and the precursor fragmentation amplitude was set to 1.0. Acquisitions were run under the control of Trap Control 7.1 software (Bruker Daltonics). All MRM data were processed using Data Analysis 4.0 software (Bruker Daltonics). The relative amounts of each target peptide were calculated as the average ratios of peak areas corresponding to the analyzed peptides. All data were manually inspected to ensure correct peak detection and accurate integration.

All analyses of plasma samples were repeated twice and standard deviations of peptides containing K525Hcy and K212Hcy, were ~20%. The interassay accuracy, determined from duplicate assays on 2 different analysis, was ≤10%.
